# Correlation of systemic involvement and presence of pathological skin calcification assessed by ex vivo nonlinear microscopy in Pseudoxanthoma elasticum

**DOI:** 10.1007/s00403-023-02557-x

**Published:** 2023-02-27

**Authors:** Luca Fésűs, Norbert Kiss, Klára Farkas, Dóra Plázár, Sára Pálla, Nastassia Navasiolava, Lili Róbert, Norbert M. Wikonkál, Ludovic Martin, Márta Medvecz

**Affiliations:** 1 Department of Dermatology, Venereology and Dermatooncology, Semmelweis University, Mária Street 41, Budapest, 1085 Hungary; 2 PXE National Reference Centre, Angers University Hospital, 4 Rue Larrey, 49100 Angers, France

**Keywords:** *Pseudoxanthoma elasticum*, Ectopic calcification, Elastin, Calcium deposit, Nonlinear optical microscopy, Multiphoton microscopy

## Abstract

*Pseudoxanthoma elasticum* (PXE (OMIM 264800)) is an autosomal recessive connective tissue disorder mainly caused by mutations in the *ABCC6* gene. PXE results in ectopic calcification primarily in the skin, eye and blood vessels that can lead to blindness, peripheral arterial disease and stroke. Previous studies found correlation between macroscopic skin involvement and severe ophthalmological and cardiovascular complications. This study aimed to investigate correlation between skin calcification and systemic involvement in PXE. Ex vivo nonlinear microscopy (NLM) imaging was performed on formalin fixed, deparaffinized, unstained skin sections to assess the extent of skin calcification. The area affected by calcification (CA) in the dermis and density of calcification (CD) was calculated. From CA and CD, calcification score (CS) was determined. The number of affected typical and nontypical skin sites were counted. Phenodex + scores were determined. The relationship between the ophthalmological, cerebro- and cardiovascular and other systemic complications and CA, CD and CS, respectively, and skin involvement were analyzed. Regression models were built for adjustment to age and sex. We found significant correlation of CA with the number of affected typical skin sites (*r* = 0.48), the Phenodex + score (*r* = 0.435), extent of vessel involvement (*V*-score) (*r* = 0.434) and disease duration (*r* = 0.48). CD correlated significantly with *V*-score (*r* = 0.539). CA was significantly higher in patients with more severe eye (*p* = 0.04) and vascular (*p* = 0.005) complications. We found significantly higher CD in patients with higher *V*-score (*p* = 0.018), and with internal carotid artery hypoplasia (*p* = 0.045). Significant correlation was found between higher CA and the presence of macula atrophy (*β* = − 0.44, *p* = 0.032) and acneiform skin changes (*β* = 0.40, *p* = 0.047). Based on our results, the assessment of skin calcification pattern with nonlinear microscopy in PXE may be useful for clinicians to identify PXE patients who develop severe systemic complications.

## Introduction

Pseudoxanthoma elasticum (PXE, OMIM#264800) is an autosomal recessive metabolic disorder characterized by progressive ectopic mineralization of elastic fibers of the connective tissues. PXE primarily affects the skin, the retina and blood vessels that may lead to blindness, coronary artery disease and peripheral arterial disease [1, 2]. Ectopic calcification is an indirect consequence of the sequence variants in the *ABCC6* gene [3, 4]. *ABCC6* encodes MRP6, a transmembrane transporter protein, with a yet unknown substrate, although its mutations result in lower serum inorganic pyrophosphate (PPi) levels. PPi is a potent inhibitor of calcification; thus, its deficiency plays a central role in ectopic mineralization in PXE [3, 5].

Cutaneous alterations present as yellowish papules that may coalesce into plaques. The first changes usually occur on the neck at the end of the first or during the second decade; though childhood onset has also been reported [6]. Later similar changes appear in the flexural areas and in the axillary or inguinal folds, and decreased skin laxity leads to the appearance of redundant skin folds [7, 8]. However, there is a wide variety of PXE regarding the onset, presentation and natural history [9].

To date, the diagnosis of PXE is mainly confirmed by skin biopsy and/or genotyping. Histopathological changes of PXE in the skin affect the mid-dermis include fragmented and clumped elastic fibers, calcium deposits and calcified elastic fibers [10, 11]. The mid-dermis contains less collagen, but elastin, microfilament and proteoglycan contents are increased. These latter macromolecules are absent from healthy elastin, and their high affinity for calcium ions leads to the accumulation of mineral precipitates [12].

Due to the huge variability of PXE phenotype, it is challenging to predict the age of onset, presentation and the disease course [13, 14]. In addition, there is no firm correlation between the severity of skin, eye and cardiovascular phenotype, with any predictors regarding the type or number of mutations [15–17]. Plasma PPi level does not correlate with the amount of mineral deposition [18]. Recently, in a pilot study we examined a small cohort of PXE patients, but found no correlation between plasma PPi levels and the Phenodex score [19]. Most studies use the Phenodex classification for severity assessment that cumulates clinical outcomes in organs affected by PXE; however, it is difficult to estimate the long-term effects of PXE and individual risk based on this system [20–22]. For clinicians, a reliable severity biomarker related to skin lesions would be useful, as skin involvement typically precedes other organs and it is easily accessible for assessment. Predicting the future outcome of PXE could improve the prevention of severe complications in those at elevated risk [6].

Recently, we have shown that molecular and structural alterations of the mid-dermis exhibited in PXE-affected skin can be visualized by nonlinear microscopy (NLM) [19]. NLM is increasingly used in dermatological research in areas such as in vivo assessment of dermal connective tissue alterations in animal studies [23, 24] and ex vivo assessment of rare diseases with skin involvement [25]. Among the different NLM modalities, two-photon excitation fluorescence (TPEF) is suitable for the visualization of endogenous fluorophores, among others, as elastin and keratin, whereas collagen generates second-harmonic generation (SHG) signal [26]. Besides SHG imaging of collagen, we successfully visualized calcification and detrimental changes to elastic fibers in formalin fixed, deparaffinized, unstained skin sections using TPEF [19].

In the present study we investigated the correlation between the clinical phenotypes and the degree of ectopic calcification in the skin using ex vivo nonlinear microscopy in a cohort of PXE patients.

## Materials and methods

### Patient data

We performed a retrospective analysis of clinical and histopathological data in patients with confirmed PXE. All patients were diagnosed and managed at the PXE National Reference Centre (MAGEC Nord), Angers University Hospital, Angers, France, as a part of the protocol for phenotyping the French PXE cohort (ClinicalTrials.gov Identifier: NCT01446380). The diagnosis was based on skin histopathology and/or detecting two mutations of the *ABCC6* gene [14]. PXE patients monitored between 2008 and 2019 were included in this study and submitted to the selection method described below. All participants gave their written informed consent for the use of their medical files for research purposes. All procedures were in accordance with the 1964 Helsinki Declaration and its later amendments. This study was approved by the Semmelweis University Regional and Institutional Committee of Science and Research Ethics, Budapest, Hungary (SE RKEB no. 193–2/2017).

### Selection of study population

Patients under the age of 18, and those where the diagnosis of PXE was not verified or where clinical data were not available were excluded from the primary evaluation. We evaluated skin biopsy slides with von Kossa and Weigert’s elastic staining of 106 PXE patients. Thereafter, patients whose samples were of lower quality, insufficient, or where von Kossa or Weigert’s elastic staining was not available were also excluded (*n* = 54). A semi-quantitative severity rating system was created for the evaluation of calcinosis based on the extent of calcinosis in the reticular dermis, excluding vessels and glands, the density and the localization of the calcium deposits. Samples were re-evaluated based on this rating system by two independent investigators and classified into five severity categories. To achieve a representative study population, with each category equally represented, five samples with the highest quality from each group were selected, a total of 25 samples.

### Medical history data collection and laboratory assessment

Medical history collected from PXE patients included age, sex, onset and duration of the disease, smoking habit, presence of diabetes mellitus, history of angina pectoris, intermittent claudication, transitional ischemic attack (TIA), stroke, gastrointestinal hemorrhage, nephrolithiasis and osteopenia/osteoporosis. Laboratory blood analysis included complete cell counts, liver and kidney function, total and low-density lipoprotein (LDL)/high-density lipoprotein (HDL), triglyceride, Lipoprotein(a) (Lp(a)), serum-glucose, HbA1C, high-sensitivity C-reactive protein (hsCRP) and vitamin D and K level measurement from fasting patients.

The Phenodex + score, as proposed by Legrand et al*.* [27], was determined for each patient where clinical data and laboratory results categorized the patient as mild (≤ 4), moderate (5–7) and severe (≥ 8) PXE. Individual components of the Phenodex + scores are referred to as skin (S), eye (E), gastrointestinal (G), vessel (V), cardiac (C) and renal (R) score.

### Cutaneous phenotype

Skin involvement was estimated by a clinical assessment of the number of affected skin sites. Ten characteristic areas: lateral and anterior sides of the neck, nape, axillary fossae, shoulder region, antecubital fossae, groin, popliteal fossae, periumbilical region, mucosal part of the lower lip, and eight nontypical areas: oblique mental creases, face, upper limbs excluding folds, lower limbs excluding folds, lumbar region, and the genital, anal and oral mucosa excluding the lower lip were examined. An area was considered as’affected,’ when at least one lesion was visible. The presence of mucosal lesions and acneiform lesions was also assessed.

### Ophthalmological phenotype

All patients underwent routine ophthalmological examination using fundoscopy. The presence of angioid streaks was determined. The presence of choroidal neovascularization, subretinal hemorrhage and scarring was assessed. Uni- or bilateral blindness and primary macular atrophy were also included among the clinical data.

### Cardiovascular phenotype

All patients underwent cardiovascular evaluation that included blood pressure measurement, electrocardiography (ECG) and echocardiography. Ankle-brachial index (ABI) was measured according to already previously established guidelines [28]. Peripheral artery disease (PAD) was defined as ABI ≤ 0.90 or > 1.40 [28]. Peripheral pulses were assessed at the lower extremities. A stenosis of the internal carotid artery and the presence of internal carotid artery hypoplasia (ICAH) were assessed with carotid Doppler ultrasound, as well as the extent of calcification of the aorta and peripheral vessels. Aortic calcification and peripheral arterial calcification—at the femoral/subpopliteal level—were classified as not present (0), minimal (1), diffuse (2) and stenosing (3), respectively, according to the ultrasound or computed tomography scan report.

### Skin samples for histopathology

Skin biopsies from PXE-affected, not sun-exposed skin sites of all patients were collected. Skin sections from the formalin-fixed and paraffin-embedded samples were stained with von Kossa (VK) stain to reveal calcium deposition, and Weigert’s elastic (WE) staining to analyze the elastic fibers. Histopathologic evaluation was performed by expert dermatopathologists.

### Nonlinear microscope (NLM) imaging and image processing

**Fig. 1:**
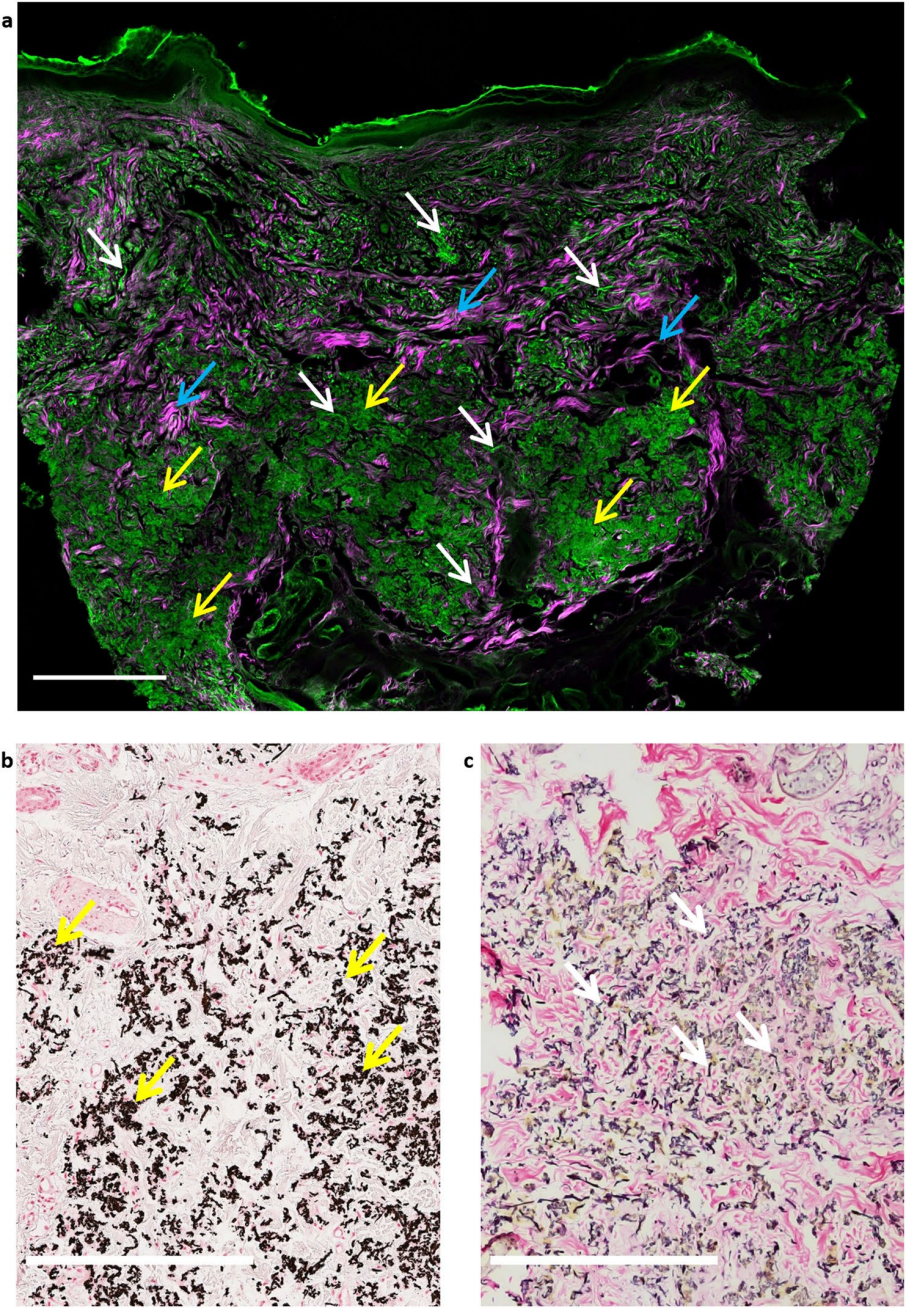
Representative two-photon excitation fluorescence (TPF) and second-harmonic generation (SHG) images of ex vivo formalin-fixed, deparaffinized, unstained PXE-affected skin sections. **a** Green (525/50 nm) emission filters were chosen to detect a high portion of TPEF signals originating from elastin and calcification in the dermis. SHG signal of collagen was spectrally differentiated with the use of a narrowband 405/20 nm filter, displayed by magenta color. Excitation wavelength was 805 nm. It should be noted that keratin also emitted TPEF signal in the green channel. Individual TPEF and SHG images of 420 × 420 μm^2^ were assembled into a two-color mosaic image. The mid-dermis contained confluent calcified areas and fragmented, clumped elastin fibers revealed by the TPEF channel. We described the same findings, when we previously compared TPEF and SHG image of PXE-affected and healthy skin [19]. **b-c** Representative histology images with tenfold magnification of the mid-dermis from the same skin sample. **b** von Kossa revealed mid-dermal calcium deposition and calcified elastic fibers. **c** Weigert’s elastic staining displayed fragmented and polymorphic elastic fibers. White arrows, elastin fibers; yellow arrows, calcium deposits; blue arrows, collagen fibers. Scale bars display 400 μm

For the NLM imaging, 20-μm-thick sections were cut and deparaffinized from the same skin biopsy blocks. NLM imaging was carried out with a ~ 20 MHz repetition rate, sub-ps Ti:sapphire laser (*FemtoRose 300 TUN LC*, R&D Ultrafast Lasers Ltd., Budapest, Hungary) operating at 800 nm central wavelength. Laser beam was focused with a 20 × water immersion objective (W-Plan—APOCHROMAT 20x/1.0 DIC (UV) VIS-IR, Carl Zeiss, Jena, Germany). To capture images, a commercial Axio Examiner LSM 7 MP laser scanning two-photon microscope system (Carl Zeiss Microscopy GmbH, Jena, Germany) with custom modified detection optics was used. Nonlinear microscope imaging experiments were performed at the Nonlinear Microscopy Laboratory of Wigner Research Institute for Physics, Budapest, Hungary. TPEF and SHG signals were collected by two NDD detectors and visualized by the ZEN 3.0 SR software (Carl Zeiss Microscopy GmbH, Jena, Germany). A 525/50 nm bandpass filter was used to capture TPEF signal of elastin and calcium deposits, and a 405/20 nm filter to collect the SHG signal of collagen. Mosaic images were acquired with a 420 × 420 μm^2^ field of view (FOV) of individual 2D images using the Mosaic stepping motor hardware and software system of R&D Ultrafast Lasers Ltd., Budapest, Hungary. TPEF and SHG images were merged and assembled into two-color mosaic images with ImageJ v1.46 software (NIH, Bethesda, MD, USA). NLM image of PXE skin is displayed in Fig. 1, together with corresponding histology images.

**Fig. 2:**
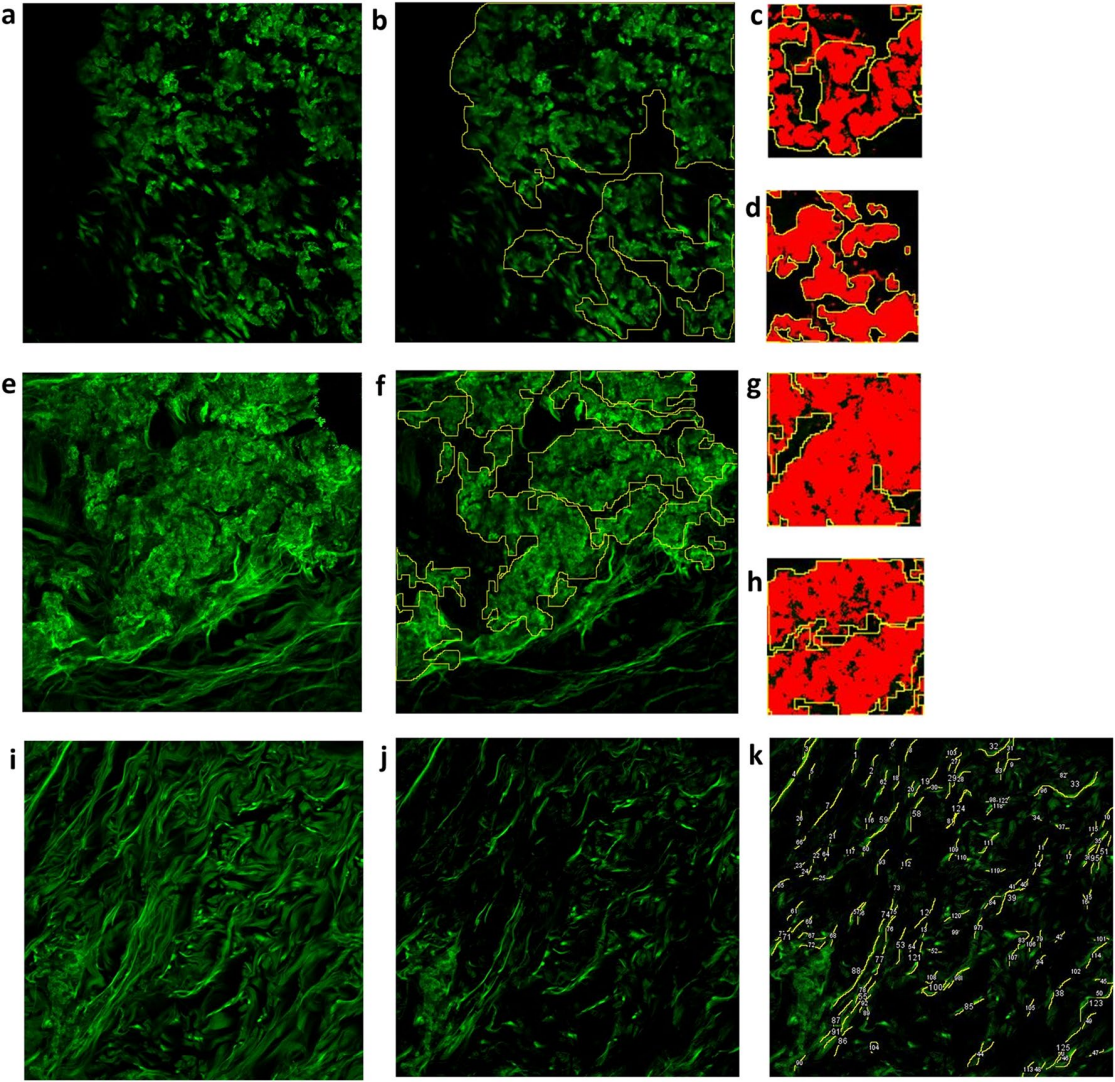
Representative two-photon excitation fluorescence (TPEF) images of the dermis of PXE patients where we illustrate how calcification area (CA) and calcification density (CD) were calculated. For each patient, ten field of views (FOV) were selected, with an area of 420 × 420 μm^2^. In case of each FOV, TPEF channel was analyzed after subtraction of second-harmonic generation (SHG) signal. **a**, **e** TPEF signal of representative FOVs after the subtraction of the SHG channel. **b**, **f** Areas of calcification were manually outlined using the ImageJ v1.46 software (NIH, Bethesda, MD, USA) to calculate CD. **c**, **d, g**–**h** Manual thresholding was applied on outlined regions of interest (ROI) to determine CD. Different CA and CD values were found in the study population. CA (23.3 ± 16.15, mean ± SD) and CD (51.6 ± 22.1, mean ± SD) showed a high SD within our PXE cohort. **a–d** Representative images for PXE-affected skin with relatively higher CA and lower CD. **e–h** Representative images for PXE-affected skin with relatively lower CA and higher CD. **i–k** Counting of elastic fiber number and length. **i** Original TPEF image, **j** TPEF image after the subtraction of the SHG channel, that was used to manually outline and number elastic fibers. **k** Number of elastin fibers in PXE patients was 40.8 ± 27.7 μm (mean ± SD) per FOV, length of elastin fibers was 32.3 [25.4 – 34.9] μm (median [interquartile range]). Image size: **a**, **b, e**, **f, i–k** 420 × 420 μm^2^; **c**, **d, g**, **h** 101 × 101 μm^2^. SD, standard deviation

In each sample, ten representative FOVs from the mid-dermis were selected for further analysis. In semi-quantitative analysis, calcium deposits were manually selected (region of interest [ROI]). To determine the relative area affected by calcification (CA), the sum of the ROIs was measured and divided by the total area of the ten FOVs. Calcification density (CD) was determined based on five ROIs of 101 × 101 μm^2^ size from each sample from calcified areas. By manual thresholding, empty areas and background noise were removed. CD was calculated as the ratio of the above-threshold and total ROI area. From these two parameters, a total calcification score (CS) was calculated by multiplying CA by CD. The number and length of elastic fibers were counted manually in each FOV using ImageJ and a mean value was calculated. Elastic fiber number is expressed as number/FOV, fiber length is given in μm unit. Calculation of CA and CD, and elastic fiber assessment are depicted in Fig. 2B. The assessment of NLM images was performed by one examiner throughout the whole study, who was blinded to the clinical characteristics of the individual patients. This study was approved by the Ethics Committee in Budapest, Hungary (SE RKEB no. 193–2/2017).

**Fig. 3:**
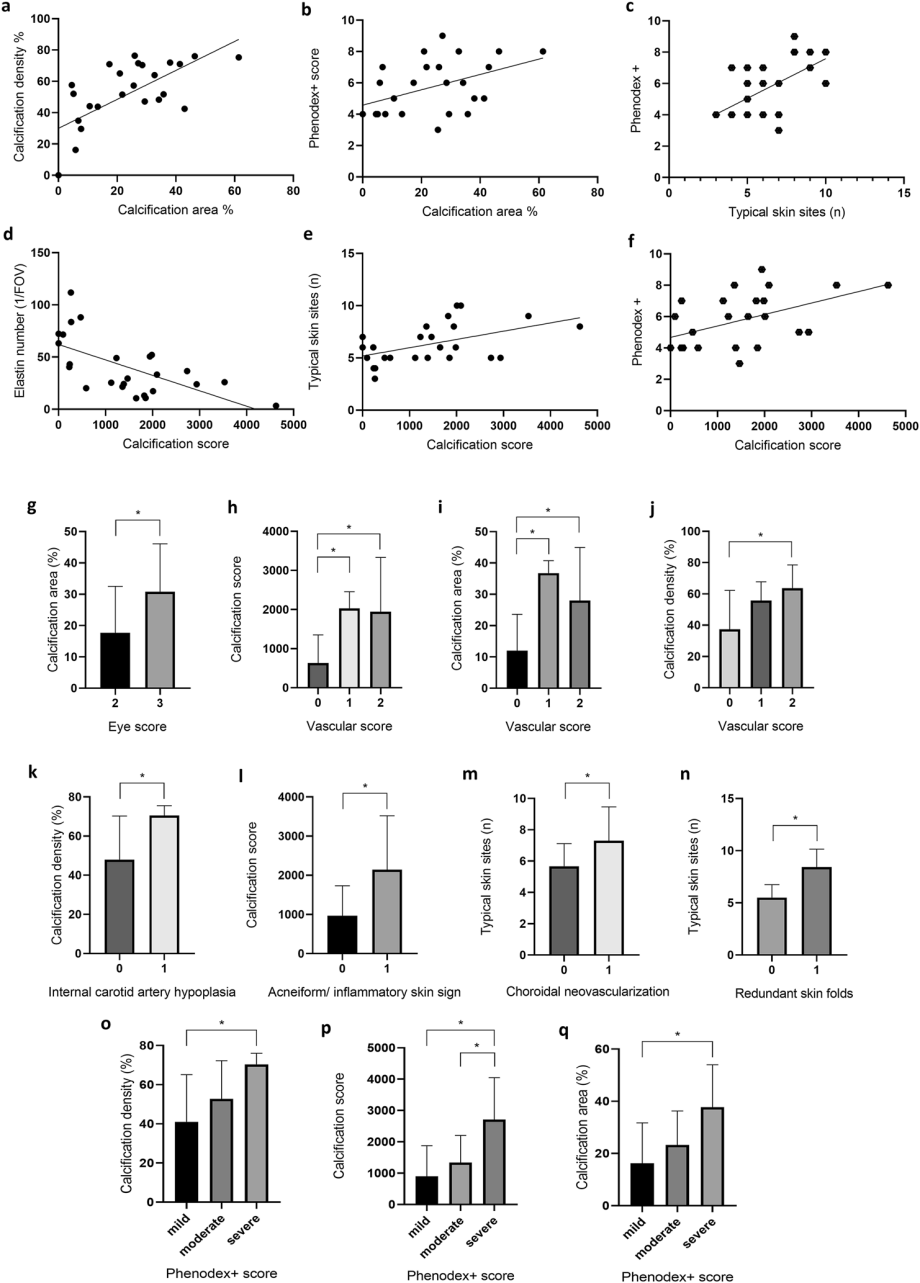
Correlation between skin calcification parameters and macroscopic skin signs or clinical phenotypes in all PXE patients. Here, statistical analyses with significant findings (p < 0.05) are represented. **a** positive correlation between calcification density (CD) and calcification area (CA), **b** positive correlation between Phenodex score and calcification area (CA), **c** positive correlation between Phenodex score and the number of affected typical skin sites. **d** inverse correlation between the number of elastic fibers (per FOV) and calcification score (CS), **e** positive correlation between the number of affected typical skin sites and CS, **f** positive correlation between Phenodex score and CS. Significantly higher CA was found in patients with more severe eye complications (E3 vs E2) (**g**) and significant differences were found in CS (**h**), CA (**i**) and CD (**j**) between patient groups with different Vascular-scores, respectively. Significantly higher CD was found in patients with internal carotid artery hypoplasia (**k**), significantly higher CS was found in patients with acneiform skin signs (**l**). **m** The number of affected characteristic skin sites was significantly higher in patients with choroidal neovascularization. **n** The number of affected characteristic skin sites was significantly higher in patients with redundant skin folds. Regarding the Phenodex + scores, significantly higher CD (**o**), CS (**p**) and CA (**q**) were found in patients with severe PXE compared to mild PXE, respectively. Statistical significance (p < 0.05) is marked with *

### Statistical analysis

Data are expressed as mean ± standard error for normally distributed continuous variables, median [interquartile range (IQR)] for nonparametric distributed continuous variables and number (%) for categorical variables. Data normality was tested with Kolmogorov–Smirnov’s test. Relationships between CA, CD, CS and the number of affected characteristic skin sites with continuous variables were examined using Pearson’s correlation coefficient (r) (or Spearman’s correlation coefficient, where appropriate). Logistic regression models were built for binary outcomes, and the Poisson regression model was built for counting data. All models were adjusted for age and sex. The Student’s *t* test (or Mann–Whitney–Wilcoxon exact test where applicable) for independent variables was performed to compare CA, CD and CS between patient groups characterized by dichotomic outcomes. CA, CD and CS were compared between patients with different S, E, V, C and R scores, distinct Phenodex + scores as well as different levels of aortic calcification and peripheral atherosclerosis with the one-way ANOVA test. A p value < 0.05 was regarded statistically significant. Statistical analysis was performed using Statistica 13.5 (StatSoft, Inc., Tulsa, OK).

## Results

### Patient characteristics

Based on the above-mentioned selection criteria, 25 patients—17 females and 8 males—were included in this study, in accordance with usual sex ratio in PXE [29]. Mean age was 49 ± 13.8 years. Among the laboratory results, the prevalence of low vitamin K levels was found in 4 (16%), hypercholesterolemia in 9 (36%) and high Lp(a) levels in 9 (36%) patients. Descriptive statistics of clinical parameters and complications of PXE as opposed to data in the literature are shown in Table 1.

**Table 1:** PXE patient characteristics

	In this study cohort	Data from the literature
Total (*n*)	25	
Age (years)	48.4 ± 13.87	31.7 [41], 47 ± 15 [42]
Male sex (*n*)	8 (32%)	32% [8], 36% [42]
Onset (age; years)	20 [14–22.5]	13.54 [43], 21 ± 14 [44]
Duration (years)	20.5 ± 15.8	
Smoking (*n*)	10 (40%)	35% [43], 30% [42]
BMI (kg/cm^2^)	24.27 (22.93–18.10)	
Osteopenia/osteoporosis (*n*)	6 (24%)	23–46% [42]
Gastrointestinal hemorrhage (*n*)	1 (4%)	18% [41]
Nephrolithiasis (*n*)	6 (24%)	39.8% [45]
Skin (*n*)		
Plaques	15 (60%)	
Redundant skin folds	7 (28%)	93% [41]
Acneiform lesions	10 (40%)	
Mucosal involvement	10 (40%)	21.4% [32]
Typical areas	6.2 ± 1.8	
Atypical areas	0 [0–1]	
CA	23.3 ± 16.15	
CD	51.6 ± 22.1	
CS	1436.0 ± 1182.5	
Elastic fiber number/FOV	40.8 ± 27.7	
Elastic fiber length (μ m)	32.3 [25.4–34.9]	
Eye (*n*)		
Peau d’orange	4 (16%)	84.9% [44]
Angioid streaks	22 (88%)	93.75% [35], 92.5% [44]
Choroidal neovascularization	10 (40%)	42.5% [35], 50.9% [44]
Bleeding and/or scarring	2 (8%)	44% [44]
Macular atrophy	5 (20%)	32% [34]
Blindness	5 (20%)	10.9% [46]
Vascular (*n*)		
Weak or absent pulses	7 (28%)	
Intermittent claudication	9 (36%)	42–53% [37]
Lower limb revascularization	0 (0%)	
ICAH	4 (16%)	8.6% [47]
Peripheral artery disease	7 (28%)	37–57% [48]
High blood pressure	4 (16%)	8–25% [37]
Stroke/TIA	1 (4%)	15% [38]
Ankle-brachial index	1.1 [0.83–1.15]	0.92 ± 0.19% [48]
Cardiac (*n*)		
Chest pain/angina/abnormal ECG	5 (20%)	13–15% [38]
Cardiac attack	0 (0%)	1–5% [38]
Phenodex + score	5.64 ± 1.8	
S	2 [1–2.5]	
E	2 [7, 36]	
G	0 [0–0]	
V	1 [0–2]	
C	0 [0–0]	
R	0 [0–0.5]	

Data is expressed as mean ± standard error for normally distributed continuous variables, median [range] for nonparametric distributed continuous variables and number (%) for categorical variables

*CA* calcification area, *CD* calcification density, *CS* total calcification score, *ICAH* internal carotid artery hypoplasia, *TIA* transient ischemic attack, *ECG* electrocardiography

### Relationship and differences between skin calcification and clinical phenotypes

Descriptive statistics of calcification parameters is found in Table 1. Correlation between calculated calcification parameters and clinical variables is shown in Table 2.

**Table 2:** Correlation between skin calcification parameters and clinical variables

	CA	CD	CS
CD	0.67	1	NS
Elastic fiber number	− 0.74	NS	NS
Elastic fiber length	− 0.42	NS	− 0.586
Onset	NS	NS	NS
Duration	0.48	NS	0.40
Visual acuity	NS	NS	NS
ABI	NS	NS	NS
Aortic calcification	− 0.44	NS	NS
Peripheral atherosclerosis	0.44	0.41	0.45
Number of affected typical skin sites	0.48	NS	0.49
Phenodex + score	0.435	0.46	0.503
S-score	NS	NS	NS
E-score	NS	NS	NS
G-score	NS	NS	NS
C-score	NS	NS	NS
V-score	0.434	0.539	0.507
R-score	NS	NS	NS

Parameters in the first column were correlated with Calcification area (CA), calcification density (CD) and calcification score (CS) of the same patient. A positive value indicates positive correlation, a negative value refers to an inverse correlation. Spearman’s *r* is presented for significant relationships. Nonsignificant correlations are marked as NS

*ABI* ankle-brachial index, *C* cardiac, *CA* calcification area, *CD* calcification density, *CS* total calcification score, *E *eye, *G* gastrointestinal, *R* renal, *S *skin, *V* vessel

CA was significantly higher in patients with more severe eye complications (E3 vs E2) (p = 0.04). We found significant differences in CA between distinct V-score groups (p = 0.005) and in CD between distinct V-score groups (p = 0.018) with the one-way ANOVA test (Fig. 3). Regarding the Phenodex + scores, significantly higher CA and CD were found in the severe than in the mild PXE patients (p = 0.036 and p = 0.027, respectively), and significantly higher CS was observed in the severe vs mild (p = 0.010) and severe vs moderate (p = 0.041) Phenodex + patients with the one-way ANOVA test, after adjustment (Fig. 3).

We found significantly higher CA among patients with redundant skin folds (p = 0.024). Also, significantly higher CD was found in the following group of patients: (1) those with ICAH (p = 0.045), (2) with plaques (p = 0.032), (3) with redundant skin folds (p = 0.033), (4) with acneiform skin signs (p = 0.039), (5) with nontypical skin site involvement (p = 0.030), (6) with weak pulses (p = 0.016) and (7) with intermittent claudication (p = 0.041). CS was also significantly higher in patients with plaques (p = 0.018), redundant skin folds (p = 0.009), acneiform skin signs (p = 0.031), ICAH (p = 0.045) and with weak pulses (p = 0.028) with the Student’s t-test. After adjustment, higher CD in patients with ICAH and higher CS in patients with acneiform skin signs remained statistically significant (Fig. 3). No differences in CA, CD and CS were found when other clinical or laboratory parameters were looked at. Additionally, with the linear regression model after adjustment three statistically significant correlations were found: higher CA and (1) the presence of macula atrophy (*β* = − 0.44, p = 0.032), (2) acneiform changes (*β* = 0.40, p = 0.047) and (3) late onset disease (*β* = − 0.48, p = 0.017).

### Macroscopic skin involvement

The number of affected typical skin sites correlated with the elastic fiber number (r = − 0.47, p = 0.018), with the age of onset (r = − 0.53, p = 0.005) and the Phenodex + score (r = 0.56, p = 0.003). After adjustment, elastic fiber number and onset lost significance. The number of affected characteristic skin sites was significantly higher in patients with CNV, (p = 0.033), redundant skin folds (p < 0.0001) and with mucosal involvement (p = 0.008) with the Mann–Whitney–Wilcoxon exact test. After adjustment, the latter one lost significance (Fig. ). Also, patients with higher S-score had higher number of affected typical skin sites (p < 0.0001) with the one-way ANOVA test. Patients with affected nontypical skin sites had a higher prevalence of CNV (pOR 5.02, 95% CI [1.33–2225.12], p = 0.02) with the logistic regression model.

## Discussion

The attempts to give insights into the natural history of PXE are still ongoing, but the number of studies that address this question is scarce [30]. Objective evaluation of the severity of PXE is challenging, although different methods were used to investigate the relationship between cutaneous and systemic involvement [18, 27, 31, 32].

We found a positive correlation between the number of affected characteristic skin site with the Phenodex + score and the severity of skin lesions, and correlation of the number of affected atypical skin sites with the presence of severe ophthalmological complications. *Utani et al.* calculated the number of affected areas with characteristic PXE lesions in six body sites. They found a higher distribution score in patients with CV disease, and wider angioid streaks [32]. A recent study revealed correlation between the number of affected skin sites and the development of severe CV and ophthalmological complications [8].

In this study, we evaluated the correlation between degrees of ectopic calcification as the major cause of the disease to the clinical manifestation. To the best of our knowledge, to date, no studies have investigated the area and density of ectopic calcification in the skin with clinical outcomes. We found significantly higher calcification area among patients with redundant skin fold. Calcification density was also significantly increased in patients affected by ICAH, plaques, redundant skin folds and acneiform skin signs. The area, density, and overall extent of calcification were significantly higher in patients with high, but not in patients with low Phenodex + scores. In the literature, a PET-CT study reported that skin and arterial ^18^F-NaF deposition, a marker for calcification, did not correlate with the Phenodex score [33]. Atrophy of the retinal epithelium is a common finding in PXE independent of CNV [34]. Although the prevalence of atrophy and CNV in our cohort was slightly lower than reported in the literature [34, 35], we found that the number of affected typical locations was significantly higher in patients with CNV and nontypical skin site involvement significantly increased the risk of CNV.

In the vasculature of PXE patients, calcification is similar to what we can observe in aging associated arteriosclerosis [36]. A highly increased rate of peripheral arterial disease (PAD) is reported in PXE [37, 38]. PAD is independently associated with higher chance of CV attacks, that could partially explain the accelerated CV aging and higher number of CV events in PXE [38]. In our cohort, the extent of aortic calcification and peripheral arterial calcification correlated with CA in the skin. Moreover, CD was significantly higher in patients with higher V-score. In population-based studies, it was shown that advanced aging exceeds conventional risk factors of the prevalence of cardio- and cerebrovascular aging [39]. Indeed, *Bartstra et al.* evoked PXE as a prime example of accelerated peripheral vascular aging [36].

PXE as a model of the heterogenous group of ectopic mineralization disorders, stands in the focus of therapeutic development. Novel therapeutic agents counteracting ectopic mineralization are tested and there are also therapeutic candidates for allele-specific therapies [40]. As ectopic calcification results in complications of PXE, it could serve as a meaningful intermediate endpoint for clinical trials [15].

In conclusion, we found a significant correlation between the area size and density of cutaneous calcification and the extent of systemic manifestations in PXE. Based on our findings, we recommend the assessment of these parameters, in addition to the evaluation of macroscopic skin involvement. The assessment of cutaneous calcification morphology may be used to manage PXE patients according to their specific risk profile. Also, it could serve as an objective parameter to monitor therapeutic efficacy in clinical trials. Whether dermal calcification pattern is suitable for predicting the progression of PXE and for the early identification of patients with more severe phenotype, needs further investigations.

## Limitations

There are limitations of this study, which necessarily inform interpretation of these results. First, despite Angers University Hospital is a PXE National Reference Centre, this is a single-center study. The study population is inclusive of a low number of adult subjects. Although PXE is a rare disease, increasing the cohort size are necessary to strengthen our results. As this was a retrospective study, it is also important to note the limitations inherent in extracting data through a medical record review process.

Next, it is important to note that here, ex vivo skin sections were examined with NLM. Applicability of the measurements to in vivo settings and interpretation of those results need further methodological and comparative investigation.

Another limitation of this study is related to the semi-quantitative analysis of the NLM images. Although image analysis was performed by the same person to exclude interobserver variability, automatization of this process may lead to more precisely comparable data.

In addition, we worked with a custom-built, laser setup and NLM measurements were carried out with the settings according to our previous works [19]. Further development of our method should include a fine-tuning of a protocolized approach to the NLM measurements.

## Data Availability

The datasets generated during and/or analysed during the current study are available from the corresponding author on reasonable request.

## Abbreviations

ABCC6: ATP-binding cassette sub-family C member 6
ABI: Ankle-brachial index
C: Cardiac
CA: Area affected by calcification
CD: Calcification density
CS: Total calcification score
E: Eye
ECG: Electrocardiography
FOV: Field of view
G: Gastrointestinal
hsCRP: High-sensitivity C-reactive protein
ICAH: Internal carotid artery hypoplasia
IQR: Interquartile range
LDL: Low-density lipoprotein
Lp(a): Lipoprotein(a)
HDL: High-density lipoprotein
MRP6: Multidrug resistance protein-6
NLM: Nonlinear microscopy
PAD: Peripheral arterial disease
PPi: Serum inorganic pyrophosphate
PXE: Pseudoxanthoma elasticum
R: Renal
ROI: Region of interest
SHG: Second-harmonic generation
S: Skin
TIA: Transitional ischemic attack
TPEF: Two-photon excitation fluorescence
V: Vessel
VK: Von Kossa
WE: Weigert’s elastic
